# Illness explanatory models of depression among young people in low-resource settings: A qualitative study in Ghana and Zimbabwe

**DOI:** 10.1371/journal.pone.0340267

**Published:** 2026-01-27

**Authors:** Denford Gudyanga, Rebecca Jopling, Moses Kumwenda, Franklin Glozah, Rufaro Mushonga, Edith Dambayi, Christopher Ayuure, Suzanne Dodd, Dzifa Attah, Arnold Maramba, Maria Anyorikeya, Lucy Owusu, Gloria Tawiah, Kenneth Adde, Fabian Achana, Raymond Aborigio, Andrea Danese, Melanie Abas, Benedict Weobong, Dixon Chibanda

**Affiliations:** 1 Institute of Psychiatry, Psychology and Neuroscience, King’s College London, London, United Kingdom; 2 Department of Pathology, Kamuzu University of Health Sciences, Blantyre, Malawi; 3 Department of Social and Behavioural Sciences, School of Public Health, University of Ghana, Accra, Ghana; 4 Department of Mental Health, Faculty of Medicine and Health Sciences, University of Zimbabwe, Harare, Zimbabwe; 5 Navrongo Health Research Centre, Navrongo, Ghana; 6 York University, School of Global Health, Toronto, Ontario, Canada; 7 The Friendship Bench, Harare, Zimbabwe; The Hong Kong Polytechnic University, HONG KONG

## Abstract

**Background:**

Depression among young people is a global public health issue, particularly common in low-resource settings like Ghana and Zimbabwe. Affecting around 10% of young people, depression often emerges between ages 15 and 24 and remains largely untreated due to stigma, limited resources, and cultural beliefs. This study explored the explanatory models of depression among Ghanaian and Zimbabwean young people, from the perspectives of young people, caregivers, healthcare workers, teachers, and community leaders, including policymakers, traditional and faith-based healers, to inform culturally relevant interventions.

**Methods:**

Qualitative interviews were conducted in Ghana and Zimbabwe. Using purposive sampling, 133 semi-structured interviews and six focus group discussions were conducted with 53 participants, including young people aged 15–24 with lived experience of depression, high school students, caregivers, community leaders, teachers, and healthcare workers. Data were thematically analysed using NVivo 14 software to identify key patterns and themes.

**Findings:**

Young people’s understanding and help-seeking behaviours reflected both emic and etic influences. Emic aspects included culturally shaped explanatory models shared by families, teachers, peers, and communities, including local idioms of distress and spiritual beliefs. Socio-economic stressors like poverty, family conflict, and academic pressure, though universally experienced, were often interpreted through culturally embedded narratives like spiritual causation, thus reflecting emic framing in participants’ accounts and influencing help-seeking in both countries. Etic aspects were evident in symptom descriptions aligning with behavioural (social withdrawal), emotional (sadness), cognitive (suicidal thoughts), and physical (fatigue) categories. Initial support was often sought from spiritual leaders, peers, and school-based resources.

**Conclusion:**

This study provides a culturally grounded understanding of how young people in Ghana and Zimbabwe conceptualise depression, shaped by both emic and etic influences. Participants described depression using local idioms and spiritual explanations, while also recognising biomedical symptoms and socio-economic stressors. These findings underscore the need for interventions that integrate traditional beliefs with evidence-based approaches. Implementing multi-stakeholder approaches that engage families, schools, and spiritual leaders may be critical in reducing stigma, improving care access, and enhancing depression outcomes for young people.

## Introduction

Depression among young people is a global public health concern with significant implications for their well-being, academic performance, and future prospects. Depression is among the typically neglected mental health conditions among young people [1], with prevalence rates ranging from 10–20% globally [2–4], and up to 15% in Sub-Saharan Africa [5]. Additionally, Jörns-Presentati and colleagues note that the prevalence of depression varies based on gender and regional sociopolitical conditions [2]. It typically emerges between ages 10 and 24, with females experiencing a 50% higher incidence than males [4]. Depression remains largely untreated in low-income countries, where 95% of those in need lack access to adequate care [6]. This results in hindered productivity, innovation, and the ability to reach educational and social potential. This is particularly critical in African countries, where 60% of the population is under 25 [7,8]. Furthermore, Fusar and colleagues [2024] highlight that the personal experiences of young people with mental health conditions like depression are often ‘siloed’ in academic studies [1]. These studies frequently lack ‘first-person’ insights into young people’s subjective lived experiences [1], potentially leading to the underrepresentation of young people in depression literature and fewer treatment interventions tailored to their unique needs.

Depression is a predominant risk factor for self-harm and suicide, which ranks as the third leading cause of death among young people worldwide [4,9]. It has been shown that depression in young people is often triggered by stressors like bereavement, relationship crises, bullying, or intimate partner violence, especially in persons with genetic or personality vulnerabilities [10–12]. Such vulnerabilities include a family history of depression, low self-esteem, and poor coping mechanisms. The impact of depression extends beyond individuals, affecting families, communities, and broader societal development. Addressing this issue requires an understanding of the psychological and social factors driving depression, particularly through culturally grounded explanatory models. These frameworks are essential for designing effective prevention intervention strategies that resonate with young people during this critical developmental stage.

The present study is informed by the Emic and Etic framework [13] and Kleinman’s Explanatory Models of Illness [14], each offering a unique perspective on how young people in low-resource settings conceptualize depression. The Emic and Etic framework distinguishes between culturally specific (emic) and universal (etic) aspects of mental health. An emic approach foregrounds local beliefs and cultural constructs, such as “*Kufungisisa”* in Zimbabwe [15–17] and “*Taamebubasugbor”* in Ghana [18], literally meaning “thinking too much”, idioms that articulate emotional and cognitive distress in ways that resonate within their respective communities. These culturally grounded idioms and expressions reflect broader spiritual explanations and social narratives that shape how young people interpret and respond to mental health challenges. Significantly influencing help-seeking behaviours, perceptions of stigma, and beliefs about the causes of distress. In contrast, an etic approach draws on standardised, cross-cultural definitions of depression, often informed by psychiatric classifications like the Diagnostic and Statistical Manual of Mental Disorders. This perspective allows for the identification of common symptom patterns across populations, such as persistent sadness, helplessness, and loss of interest. By integrating both emic and etic perspectives, this study examines how Ghanaian and Zimbabwean young people, along with other key stakeholders, construct explanatory models of depression that are grounded in their lived experiences and shaped by their specific cultural and social contexts. This dual approach not only respects the cultural specificity of depression experiences but also facilitates dialogue between local knowledge systems and global mental health frameworks, an essential step toward developing culturally responsive interventions.

Explanatory models, as conceptualized by Kleinman et al. [1978], describe individuals’ beliefs about illness, encompassing the personal and social meanings they associate with causes, symptoms, and their expectations regarding help-seeking behaviour [14]. These models differ from and complement the biomedical understanding of ‘disease,’ which focuses on the physiological and psychological processes within an individual. Instead, explanatory models emphasise the lived experience of illness, which is heavily shaped by cultural and societal contexts. This distinction is especially important in settings where cultural beliefs influence how health and illness are understood, diagnosed, and treated. For example, when healthcare professionals address a disease purely from a biomedical perspective without considering patients’ cultural beliefs or explanatory models, patients may disengage from care or not adhere to treatment regimens. Understanding these models is important to fostering culturally sensitive care that aligns with patients lived experiences and treatment expectations, thereby improving outcomes. As Fusar-Poli and colleagues [2024] argue, the early onset of depression, compounded by environmental and biological factors, exacerbates the already ‘high personal burden’ of depression, leading to subpar outcomes and even reduced life expectancy of up to 10–15 years [1].

In African contexts, explanatory models of depression among adults often revolve around socioeconomic stressors and sociocultural beliefs [16,19]. Additionally, supernatural explanations for depression, such as spirit possession or witchcraft, are common in many African cultures [20,21]. For example, in Ghana, traditional and faith healers often attribute mental health conditions, including depression, to supernatural causes, shaping how individuals perceive their illness and seek treatment [22,23].

While these cultural beliefs have been well documented in adult populations [19], youth-specific models warrant distinct attention due to developmental, social, and generational differences. Adolescents and young adults are navigating identity formation, peer dynamics, academic pressures, and transitions into adulthood, all of which shape their experiences and interpretations of mental distress. Moreover, young people may be more exposed to formal education, digital media, and global discourses on mental health, which can influence their explanatory models in ways that diverge from older generations. These factors may result in hybrid models that blend biomedical language (e.g., “stress,” “mental health”) with culturally embedded idioms and spiritual beliefs. Understanding these unique perspectives is essential for designing interventions that are developmentally appropriate, culturally resonant, and acceptable to young people themselves. Especially as emerging studies suggest that the cultural and social contexts shaping depression among African young people are distinct from those of adults.

The present study addresses this gap by incorporating diverse perspectives, including those of caregivers, healthcare workers, policymakers, teachers, and young people themselves, to contribute to the development of culturally relevant interventions that align with young people’s needs and beliefs. In doing so, it seeks to ensure that interventions for youth depression are both culturally sensitive and impactful [19]. Specifically, this study examined how young people in Ghana and Zimbabwe understand depression, its causes, symptoms, and treatment, drawing on insights from multiple stakeholder groups to inform the design of targeted mental health interventions.

This study forms part of the formative phase of the African Youth in Mind consortium, a broader programme aimed at adapting and testing the feasibility and acceptability of task-shifted interventions for youth depression in Ghana and Zimbabwe [24]. The intervention builds on the Friendship Bench model [15], which employs trained lay health workers to deliver evidence-based psychological support, such as problem-solving therapy, within community and educational settings. Task-shifting is particularly relevant in low-resource contexts where specialist mental health services are limited. This formative study, therefore, provides essential data to ensure that the adapted interventions resonate with young people’s explanatory models of depression, reflect local idioms of distress, and are acceptable to young people and key stakeholders, including caregivers, educators, and community leaders.

## Methods

### Study design and sample

Using a cross-sectional qualitative research design, a total of 186 key stakeholders in the Upper East Region of Ghana and Harare Province of Zimbabwe participated in semi-structured interviews (n = 133) and six focus group discussions (FGD) (n = 53) to reach data saturation. 109 participants were from Ghana and 77 from Zimbabwe. A purposive sampling technique was employed to recruit participants who were most likely to provide relevant and diverse perspectives on depression in young people. This included interviews with young people with lived experience of using mental health services for depression (n = 27), school-going young people not using mental health services (n = 43), caregivers (n = 14), school staff (n = 39), healthcare workers (n = 22), traditional and faith-based healers (n = 6), and policymakers (n = 15).

The FGDs involved 53 participants: 33 young people in Ghana and 20 lay health workers in Zimbabwe to capture diverse perspectives. In Zimbabwe, two FGDs were conducted, each with 10 Friendship bench lay health workers who were all female. Four FGDs were conducted in Ghana with students with no history of receiving mental health services. Two groups were made up of male students, and the other two were made up of females. Of the male groups, one group was conducted in a single-sex (male) school and one single sex (female) school. The other two groups were in mixed (both male and female) schools. In Ghana, FGD participants were composed as follows: two FGDs had 9 Female high school students each, the third FGD had 9 male students, and the fourth FGD had 7 Male students. Table 1 provides the demographic breakdown of participants by age range, gender, and stakeholder group.

**Table 1:** Demographic characteristics of respondents (n = 186).

Participant Category	Ghana					Zimbabwe					Total
	N	Gender		Age		N	Gender		Age		
		F	M	Min	Max		F	M	Min	Max	
Young People Using Mental Health Services	12	8	4	15	18	15	7	8	17	23	27
Young People Not Using Mental Health Services	33	17	16	15	18	10	5	5	17	23	43
Care Givers	4	3	1	35	70	10	9	1	40	80	14
School staff	26	6	20	18	59	13	9	4	18	58	39
Health Care Workers	18	10	8	27	49	4	4	0	43	60	22
Lay Health Workers	0	0	0	0	0	20	20	0	22	27	20
Traditional – Faith-based healers	6	3	3	37	75	0	0	0	0	0	6
Policy Makers	10	3	7	32	54	5	3	2	35	51	15
TOTAL	109	50	59			77	57	20			186

This approach was appropriate given the study’s aim to examine how young people in Ghana and Zimbabwe understand depression, its causes, symptoms, and treatment, through diverse stakeholder perspectives, to inform targeted depression interventions. Participants were selected based on their direct experience with, or involvement in, youth mental health, as outlined in the study information sheets and consent forms provided to them. Purposive sampling allowed for the inclusion of individuals who could offer in-depth, context-specific insights into both the health system and sociocultural factors influencing mental health care. This ensured that the data captured was rich, relevant, and directly informed the development of culturally appropriate depression interventions for Ghanaian and Zimbabwean young people.

The study was conducted from December 2022 to May 2023 and was part of the formative work to adapt a task-shifted intervention to treat depression for young people aged 15–18 in Ghana and 15–24 in Zimbabwe. The age range for young people differed between Ghana and Zimbabwe due to contextual and ethical considerations. In Ghana, recruitment was limited to high school students within the Navrongo Health and Demographic Surveillance Site, where typical ages for high schoolers ranged from 15–18 years, and the adapted intervention being informed by this study would primarily be school-based. In Zimbabwe, the inclusion of young people up to age 24 was informed by the Friendship Bench programme’s existing engagement with youth in this broader age range, and their intervention would be provided in primary health care settings for older adolescents and young adults. While this difference may affect direct cross-country comparability, thematic analysis focused on shared explanatory models and help-seeking behaviours across the adolescent–young adult spectrum, allowing for meaningful synthesis of findings to inform the adaptation of the Friendship bench model to develop an intervention for young people with depression.

### Study setting

Data were collected at study sites in Harare, the capital city of Zimbabwe. These included primary care clinics, government-run high schools (mixed-gender status), and the Friendship Bench implementation sites. The Friendship Bench is a private voluntary organization that provides mental health services for depression in Zimbabwe. In Ghana, data were collected at study sites within the Navrongo Health and Demographic Surveillance Site (NHDSS) area, under the Navrongo Health Research Centre (NHRC) of the Ghana Health Service. These included primary health care clinics and senior high schools situated in or near Navrongo. Navrongo is the capital of Kassena-Nankana municipality in the Upper East Region of northern Ghana, adjacent to the border with Burkina Faso. These institutions were selected based on accessibility, existing partnerships, and their relevance to youth mental health service delivery due to the high prevalence of common mental disorders.

## Participants and procedures

### Inclusion/exclusion criteria

#### Semi-structured interviews.

A mix of young people, including high school students and those with lived experience of depression, were included if they met the criteria of being aged between 15–18 in Ghana and 15–24 in Zimbabwe and had either received care for symptoms of depression or were attending high school. Caregiver consent for those aged 15–17 years was an inclusion criterion in both countries. Young people with lived experience of depression were excluded if they had an active major mental disorder, advanced physical illness that would interfere with their ability to take part in the study, or were actively suicidal, as assessed through their healthcare provider.

Key stakeholders were included if they met the criteria of being a) individuals working to deliver or implement mental health services in Zimbabwe and Ghana, b) individuals in a position of authority within the community setting (community gatekeepers), or c) primary caregivers or guardians of young people aged 15–18 (Ghana) and 15–24 (Zimbabwe), were also aged 18 and over, and willing and able to take part in the study. Caregivers and guardians of young people had to be aware that the young person was receiving care for depression (assessed through discussion with the young person before approaching the caregiver). Individuals working to deliver or implement mental health services for young people were excluded if they had not been working in the role for 12 months or more.

#### Focus group discussions.

Young people were included in Ghana if they met the criteria of being aged between 15–18, attending senior high school within the study area, and willing to take part. And in Zimbabwe, if aged between 15–24, attending senior high school, as well as willing and able to take part.

### Procedures

Potential participants were identified through their work, school attendance, community roles, or access to depression-related health services. In Zimbabwe, the Directors of Mental Health and School Psychological Services, and in Ghana, Municipal and District Directors of Health and Education, were first approached as gatekeepers and recommended other key stakeholders. Letters were sent to heads of public and private schools, and community gatekeepers were identified using national and local records. While this approach facilitated access to diverse stakeholder groups, it may have introduced selection bias by favouring individuals already engaged with formal systems or known to service providers. To mitigate this, we used purposive sampling to ensure inclusion of participants with varied experiences, including those with limited prior engagement with mental health services. We also worked with local community leaders and youth representatives to identify participants outside formal institutional settings, to enhance representativeness and contextual relevance.

In Zimbabwe, young people aged 15–24 with lived experience of poor mental health were identified through the Friendship Bench intervention (a Zimbabwean intervention for depression). They were first contacted by their care providers to gauge interest in the study and, if interested, were referred to a local research assistant for informed consent procedures. In Ghana, young people aged 15–18 were identified through healthcare centres in schools and clinics, screened for eligibility by healthcare workers, and contacted by their usual providers to assess interest. Caregivers were identified with the permission of the young people receiving mental health services.

All participants were invited to participate in the study using a standardised information sheet by trained research assistants. To mitigate any pressure for stakeholders to participate, the voluntary nature of participation was highlighted. Students and those engaged in health services were assured that declining participation in the study would not result in losing privileges as students or any change to their health care.

The interviews were conducted by research assistants (RA) (five males and two females). In Zimbabwe, two RAs were male and one female. In Ghana, three Ras were male, and one was female in private ad safe spaces. The RAs in both Ghana and Zimbabwe had a minimum of a bachelor’s degree in social sciences and had prior experience in qualitative research methods. The RAs had no prior relationship with the participants before the interviews and were trained in the topic guides, study protocol, and good clinical practice. The RAs in Zimbabwe were bilingual in Shona and English. The RAs in Ghana were multilingual in Kasem, Nankani, and English. Given the sensitive nature of mental health topics, especially among young people, we considered gender dynamics during recruitment and interviewing. While full gender-matching was not always feasible due to staffing constraints, efforts were made to ensure that participants felt comfortable. For example, female RAs were assigned to interview female students where possible, and all RAs received training on creating safe, non-judgmental spaces. Participants were also informed that they could request a different interviewer or withdraw at any time without consequence. In cases where distress was observed or reported, participants’ safeguarding standard operating procedures were followed and overseen by the study team’s clinical psychologists, who were on hand to offer psychological support if needed. As well as referral to other appropriate support services, like school counsellors or other healthcare providers. These procedures were designed to ensure participant safety and emotional well-being throughout the study.

#### Topic guides.

Topic guides were informed by a situational analysis of youth mental health in Ghana and Zimbabwe and designed to explore explanatory models of depression and perceived needs for youth mental health interventions. Their development drew on literature on youth mental health, help-seeking behaviours, and intervention delivery in low- and middle-income settings, guided by the Emic/Etic framework to balance culturally specific constructs with clinically recognised symptoms. Input from local stakeholders, including mental health practitioners, researchers, and youth representatives, ensured contextual relevance and clarity.

The guides were piloted with a small sample of young people and key stakeholders in both countries, and feedback informed key refinements. Language was simplified for younger participants, culturally salient probes were added, and the sequencing of questions was adjusted to build rapport before discussing sensitive topics. In Ghana, questions on spiritual beliefs were expanded to reflect local explanatory models. These revisions enhanced the guides’ cultural sensitivity, contextual appropriateness, and developmental suitability.

#### Data analysis.

Thematic content analysis guided the interpretation of the data. Recorded interviews were transcribed verbatim and, where necessary, translated from Shona or Kasem/Nankani into English immediately after each interview by bilingual research assistants who were native speakers of the respective languages. To ensure fidelity to original meaning, especially for culturally embedded emic terms, translations were reviewed by senior researchers familiar with local idioms and mental health terminology. Although back-translation was not feasible due to resource constraints, accuracy checks were performed through team-based review of selected transcripts, and discrepancies were resolved collaboratively to maintain semantic integrity.

An initial codebook was developed using both the topic guides and the first set of interview transcripts. NVivo Collaboration Cloud (Version 14) was used to code and organise the data. Each transcript was assigned to at least two coders, who independently read and familiarised themselves with the content. Coders added new codes inductively where appropriate. These were reviewed and merged at regular intervals using NVivo’s collaboration function to ensure consistency across the coding team. Data saturation was assessed iteratively during the coding and analysis process. The research team utilised bi-weekly meetings to review emerging themes and determine whether new interviews were yielding novel insights. Saturation was considered reached when no new codes or themes were identified in subsequent transcripts, and existing categories were sufficiently populated with diverse participant perspectives.

The Emic/Etic framework and Kleinman’s explanatory models of illness informed both the coding process and the interpretation of themes. Emic codes captured culturally specific expressions, beliefs, and idioms of distress, while etic codes reflected broader, cross-cultural constructs of depression and mental health. This dual lens allowed for a nuanced understanding of how participants made sense of their experiences, balancing local meaning systems with globally recognised symptom patterns. Kleinman’s framework further guided the identification of explanatory models, helping to distinguish between perceived causes, symptoms, and help-seeking behaviours.

A final round of coding was conducted to ensure specificity and consistency across the dataset. A team of at least three researchers, including one senior researcher (MK and FG), collaboratively reviewed the coding framework and finalised the thematic structure.

#### Ethical approval.

The study was approved by the Medical Research Council of Zimbabwe (Ref: MRCZ/A/2965), King’s College London (Ref: HR/DP-21/22–32917), Ghana Health Service (Ref: GHS-ERC: 026/07/22), and Navrongo Health Research Centre Institutional Review Board (Ref: NHRCIRB480).

## Results

While the study included a diverse group of stakeholders, including caregivers, lay health workers, traditional and faith-based healers, and policymakers, the Results section primarily focuses on narratives from young people, teachers, and school-based health professionals. This emphasis reflects the richness and volume of data from these groups, particularly regarding explanatory models and help-seeking behaviours. Perspectives from other stakeholder groups were more limited or context-specific and are therefore referenced selectively. For example, traditional healers often attributed depression to spiritual causes, such as ancestral displeasure or witchcraft, while policymakers highlighted systemic gaps in youth mental health services. These insights were incorporated into the thematic framework and informed the interpretation of findings, even if not all were presented in direct quotes.

**Fig 1:**
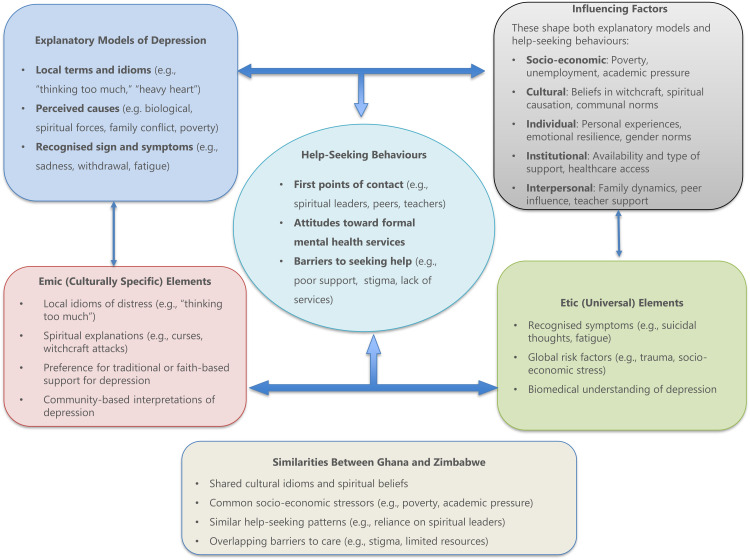
Conceptual framework illustrating Kleinman’s explanatory model of illness and emic/etic influences on youth depression: Insights from Ghana and Zimbabwe.

We identified two main constructs of depression among young people in Ghana and Zimbabwe: (a) explanatory models of depression, which include understanding its causes, signs, and symptoms, and (b) help-seeking behaviours, such as the first point of call when seeking help and attitudes towards available support (see Fig 1). These findings are presented in line with Kleinman’s Explanatory Model of Illness, which provides a framework for understanding how individuals and communities interpret illness through culturally shaped categories.

Young people’s explanatory models reflected both personal experiences and beliefs, as well as those shaped by influential figures in their social environments, including family members, teachers, peers, and community leaders. The Emic/Etic framework further informed the analysis: emic perspectives were evident in culturally grounded explanations of depression, such as spiritual causes or social disharmony, while etic elements emerged in references to biomedical symptoms and clinical terminology. These socially mediated beliefs significantly influenced help-seeking behaviours, shaping whether young people turned to informal, community-based support systems or formal mental health services.

### Explanatory models of depression

#### Understanding of depression.

The understanding of depression among young persons in Ghana and Zimbabwe reveals a multifaceted perception of the condition. Participants described it as a profound sense of sadness and loneliness, often accompanied by feelings of worthlessness and isolation, highlighting the intense emotional and physical experiences associated with depression. These descriptions reflect both emic interpretations, rooted in local expressions of distress, and etic elements aligned with clinical symptomatology. In line with Kleinman’s Explanatory Model of Illness, these understandings were shaped not only by personal experiences but also by cultural meanings attached to emotional suffering. To emphasise the emotional aspect of depression, one young person from a Ghana focus group discussion (FGD) with senior high school girls shared that:

*Depression is the mood of a person or a disorder that causes sadness*. (Ghana FGD with high school girls’ participant)

Additionally, feelings of being misunderstood and alienated were common, as another participant noted:

*Talking of depression, you feel you are left alone, you feel the whole world is against you, and you really do not appreciate who you are.* (Ghana FGD with high school girls’ participant)

The psychological impact of depression was also evident, with descriptions such as:

*Depression is a psychological feeling of sadness, you do not feel well about yourself, you look down upon yourself, you do not feel happy, there are things people do, and you think like, you cannot move further.* (Ghana FGD with high school girls’ participant)

The above sentiment was echoed by another focus group participant who stated:

*Depression means a feeling of being extremely unhappy, that is how I understand depression.* (Ghana FGD with high school boys’ participant)

Furthermore, participants shared some behavioural manifestations of depression like the way psychosis is conceptualised, with one young person describing it as:

*A depressed person behaves as if he is mad, but he is not mad.* (Ghana FGD with high school boys’ participant)

The above sentiment highlights the stigma and misunderstanding surrounding depression and, more broadly, the understanding of mental health conditions in general, which are often seen as manifestations of madness. Another young person elaborated that:

*This* [depression] *is a medical situation when one feels lonely, sad, and unhappy about his or her life. At other times, they always behave awkwardly, and waywardly and people would be thinking that their nerves are not working anymore but the other way is the situation that they are in that always makes them feel that way.* (Ghana FGD with high school boys’ participant)

In Zimbabwe, the understanding of depression centred around rumination or commonly characterised by unhealth-obsessive like thinking about one’s problems. One young person stated that:

*I think depression is when one is overthinking about life issues or thinking deeply about life, or what you are currently experiencing, which will lead to depression.* (Young person with mental health lived experience, Zimbabwe)

Another added:

*To my own understanding, depression is a stage where you’re stuck in your own thoughts. You are over assuming everything you’re thinking about, and you overload yourself with those thoughts, and you feel like there is no help you can get from anybody, and you cannot even help yourself.* (Young person with mental health lived experience, Zimbabwe)

These insights reflect the pervasive and debilitating nature of depression as experienced by young people in Ghana and Zimbabwe.

#### Beliefs and perceptions about the causes of depression.

The causes of depression among young people in Ghana and Zimbabwe were shown to be multifaceted, encompassing the school environment, difficult life circumstances, and biological factors. Participants emphasised that these causes were closely tied to emotional struggles, often rooted in broader familial and societal challenges such as family conflict, the breakdown of homes, and persistent financial insecurity (see Table 2). These findings reflect emic understandings of distress, shaped by lived realities and local interpretations of suffering, while also aligning with etic perspectives that recognise structural and biological contributors to mental health. In line with Kleinman’s Explanatory Model of Illness, these beliefs illustrate how young people make sense of depression through both personal and culturally embedded lenses, where social adversity is seen as a key driver of emotional distress.

**Table 2:** Causes of depression in Ghanaian and Zimbabwean young people.

Causes of depression	
Category	Themes
**Community issues**	Early/ forced marriages, Stigma, Gender norms
**Biological**	Inherited from family, an Accident at birth
**Challenges at home**	Lack of support from parents, Divorce or Death of parents, coming from a Broken home, Abuse at home: Physical and Emotional Abuse, Restricting food
**Individual Level**	Alcohol and drug abuse,
**School level**	Academic pressure
**Financial difficulties or Poverty**	Unemployment

#### Community issues.

Young people, especially girls, identified early and/or forced marriages as a significant societal issue contributing to depression among adolescent girls in Ghana and Zimbabwe. This was often compounded by the breakdown of such marriages or pressure from family expectations to remain in them, including cultural norms surrounding female sexuality. Additional stress arose from the need to balance marriage or pregnancy with school responsibilities. These experiences reflect emic understandings of distress rooted in gendered cultural expectations and social obligations, while also aligning with etic perspectives that recognise structural and psychosocial stressors as key contributors to adolescent mental health. In line with Kleinman’s Explanatory Model of Illness, these accounts illustrate how community-level factors shape both the perceived causes of depression and the lived experience of emotional suffering.

*If you* [as a guardian] *force a young girl into marriage, she would be thinking that maybe you dislike her, and it will not even make her to be happy at home and if it happens like that it will make the person to be depressed.* (Ghana FGD with high school girls’ participant)

*From the cultural angle, I think marriage can be the cause of depression. This is usually on the part of the girls even in the modern society some girls used to marry at a young age even before they complete junior high school. If they thought of you getting married to someone, it might disturb you a lot and might cause depression.* (Ghana FGD with high school boys’ participant)

Another participant showed how the prospects of getting into a forced marriage resulted in a young people almost taking their own life. Highlighting the pervasive effects of family poverty coupled with cultural beliefs on the role of arranged early marriage to escape poverty. FGD participant shared that:

*There was one girl who was attending school and then because the parents were not rich enough and there is one man who is very rich, so he was trying to push the girl into marriage and because the girl wants to go to school and be someone in future, the girl remained* [confined herself] *in the room and nearly killed herself and the friend rushed in to help her.* (Ghana FGD with high school girls’ participant)

Another participant explained how some traditional practices around female sexuality could be contributing to depression among young people who are made to undergo cultural rites of passage like female genital mutilation.

*You see back in those days; they used to have something called the circumcision of the female genital parts.* [In] *this modern Ghana, some of the tribes have stopped this practice but some of the tribes are still doing it. And if you are a female child and you are in that society and maybe they come around and said is time for you to cut parts of your genitals, you will be feeling afraid because you have heard something that if they cut your genitals, you can bleed till you die. So, you will be afraid to submit yourself to them to cut your genitals.* (Ghana FGD with high school boys’ participant)

Stigma and discrimination also came out as one young person in Zimbabwe shared how people living with HIV are discriminated resulting in their getting depressed.

*It is like, you’re on ART treatment* [HIV medication], *and you visit your aunt’ place, you would be given your own spoon or cup. You would feel bad around other children, so this would lead you into depression or stress.* (Young person with mental health lived experience, Zimbabwe)

#### Biological causes of depression.

Biological factors were also mentioned, including accidents at birth and inherited mental health conditions. As one young person explained:

*Some of them it seems like when they give birth to them, they can give birth to somebody, and the person can fall down. Some that can affect the brain.* (Young person with mental health lived experience, Ghana)

#### Family-related problems as causes of depression.

Family dynamics and abuse were also highlighted as significant contributors to depression in young people. One participant recounted that:

*Those who say they’re depressed is because they would be being violated/abused. A kid on our next-door, stays with a stepmother, even own (step) mother abuses the child to the extent that she asks the child to imitate whatever she does, and the child does as told, and some other time the child is deprived of food/starved the whole day.* (Young person with mental health lived experience, Zimbabwe)

Broken homes and witnessing parental conflicts were also brought out as significant stressors, causing depression in young people.

*Okay, for example, like the broken homes, let’s say your mother leaves the house and you, your siblings, and your dad. You know your father can’t take proper care of you people and if you are the elder one in the house, you will be worried as to what to do to help your younger ones because your father will not help.* (Young person with mental health lived experience, Ghana)

*The moment l see my mother and father fighting early in the morning before l go to school, l am going to be mentally disturbed.* (High school student, Zimbabwe)

A high student in Zimbabwe shared their observations that:

*Being ill-treated by parents, they may be staying with a stepmother, sometimes they may be denied food, asked to do too much work, sometimes they can be told to not to go to school these are the things that happen in life that can affect the mind.* (High school student, Zimbabwe)

Participants also highlighted how a vicious cycle of poverty and broken romantic relationships, particularly when pregnancy or children are involved as a result of such relationships, can sometimes be at the root of depression in young people. One participant shared their lived experience, saying:

*We grew up as poor children and we did not go to school. So, when we grew up, our life remained the same. We’re poor and we rent. And right now, I have a child, and that child does not have a father, and the father denied him* [refused responsibility and or denied paternity]. *So that is what depresses me a lot about our lives.* (Young person with mental health lived experience, Zimbabwe)

The loss of a loved one was another profound cause of depression, affecting young people not only through grief and trauma but also through the loss of financial support, particularly when the deceased was the family’s primary breadwinner. These experiences reflect both the emotional and structural dimensions of distress, aligning with Kleinman’s Explanatory Model of Illness, which recognises how illness is shaped by both personal suffering and broader social realities. Emic interpretations often framed such loss in deeply relational and spiritual terms, while etic perspectives highlighted the psychological and economic consequences. High school teachers shared how deeply affected young people are, noting that:

*Some of them, when they lose a loved one, a parent. That child is quite difficult to bring them back* [difficulties motivating them to engage in academics], *and others just shut down, for you to get through to them… others we can even lose them.* (High School Teacher, Zimbabwe)

*There’s also neglect, issues to do with parental loss, the death of a parent, which takes a toll on the child.* (High School Teacher, Zimbabwe)

Echoing the same sentiments as the teachers, a focus group discussion participant in Ghana said:

*for instance, being in school like this, you hear that one of your relatives is dead. You will always be depressed, and you will want to go back home.* (Ghana FGD with high school boys’ participant)

#### Individual-level causes of depression.

Participants shared that there is a growing trend of drug abuse among young people that could potentially be the cause of depression due to their effects on the body. It was also noted that some young people are abusing drugs due to peer pressure, and taking drugs could be an attempt to fit in with their peer group. From an emic perspective, these explanations reflect local understandings of depression rooted in social belonging and bodily harm. Using Kleinman’s model, these views highlight culturally grounded beliefs about aetiology and social context, contrasting with more etic, biomedical interpretations of depression.

*Some are abusing drugs just because of peer pressure thereby leading to mental health disturbance.* (High school student, Zimbabwe)

*Alcohol and drugs, like when you take alcohol, it can affect your system, and you will be living abnormal way.* (Ghana FGD with high school girls’ participant)

*The cause I also know is drug abuse. Some of the drugs if you take them, it might damage your brain.* (Ghana FGD with high school boys’ participant)

*We have seen a lot of cases at this school of students being caught with drugs and most of them are having suicidal thoughts.* (High school student, Zimbabwe)

#### School-related causes of depression.

Young people shared that a significant source of distress stems from concerns about how to sustain their educational pursuits, particularly in situations where the family breadwinner has passed away or where divorce has fractured the family unit. For example, within the school environment, stressors like pressure from family on academic expectations or the inability to pursue one’s preferred educational interest were identified. These insights reflect emic understandings of depression, where educational instability and family disruption are seen as key stressors. Through Kleinman’s lens, such narratives emphasise locally meaningful causes and social suffering, which may not align with etic, clinical models that prioritise individual pathology. As one participant noted:

*Some students are always forced to come and do some courses they do not want to do, and, in that case, it affects you because it is not your strong area.* (Ghana FGD with high school girls’ participant)

Another young person mentioned how poor school performance despite putting in extra effort is affecting high school students.

*The person normally compares himself or herself to his or her colleagues. The way they normally perform, and he is not able to perform that upon his or her hard work. So poor* [school] *performance always leads to that* [depression]. (High school student with mental health lived experience, Ghana)

This shows how the pressure to excel academically and the constant comparison with peers can lead to significant distress among students. These challenges within the school environment are a major source of stress for young people. Furthermore, the fear of disappointing parents with poor academic results adds another layer of concern, as highlighted by one teacher:

*Then, as we draw near the end of the term, the students will take home their report books, others are scared about getting home with a bad report, fear grips them, how will they present such a report to their parent and how their parent would react.* (High School Teacher, Zimbabwe)

Bullying was another important factor argued to be causing distress to young people within the school environment, as one young person puts it:

*Situations such as when we here at school, one can succumb to bullying. So that person ends up bottling everything and they can’t share it with anyone.* (High School student, Zimbabwe)

Another Zimbabwean high school student echoed that:

*When you are being bullied at school, you lose self-esteem, confidence, and you get depressed.* (High School student, Zimbabwe)

#### Difficult life circumstances as causes of depression.

Difficult life circumstances, particularly financial struggles, played a significant role in causing depression among young people. These challenges highlight a complex mix of factors beyond their control that contribute to depression. From an emic perspective, such lived experiences reflect culturally grounded understandings of distress, where economic hardship and social instability are central. Kleinman’s model helps frame these narratives as expressions of social suffering, which may be overlooked in etic, biomedical approaches. To be effective and contextually relevant in Ghana, Zimbabwe, or similar settings, interventions or programs aimed at treating depression in young people will need to address these complex issues. One young person shared:

*As you are in school, they will say buy book* [for use in class activities], *book is sixty cedis* [Ghanian currency]*, who are you going to call to get sixty cedis, you don’t have anyone to call and get sixty cedis, so it makes you to be worried.* (Ghana FGD with high school girls’ participant)

Another participant described the impact of poverty on their mental health saying:

*The food is not sufficient enough to sustain me on campus and I do not receive money from home to buy food, and also to pay for extra classes tuition, and buying notebooks is a problem. Sometimes, when I am always in bed worried and thinking, I don’t always sleep* (Young person with mental health lived experience, Ghana)

Similar sentiments were echoed by a focus group discussion participant in Ghana sharing that:

*My own* [problem] *is lack of financial support. Like we all here are students in the school and you will come and meet your colleagues and then your colleagues are showing you gari* [Ghanaian food], *and then those sort of foodstuff and when you go and open your chop box* [personal food trunk for boarding school students], *you see nothing so you feel like why am I the only poor person in the world, why is it that I cannot even purchase some of those things.* (Ghana FGD with high school boys’ participant)

### Signs and symptoms of depression

Signs and symptoms of depression were grouped into behavioural, mental, physical, and emotional/feelings classifications (see Table 3).

**Table 3:** Signs and symptoms of depression in Ghanaian and Zimbabwean young people.

Signs and Symptoms of depression	
Category	Themes
**Behavioural**	Isolation: Social withdrawal, Sleep: Sleeping too much, unable to sleep. Absent-mindedness, Talking to oneself, Crying
**Feelings**	Anger, Fear, Loss of Interest, Sadness, Worried, Mood swings
**Thoughts**	Unable to concentrate, Thinking too much, Deep thoughts
**Physical symptoms**	Falling or fainting, Appearance: including weight gain or loss. Lack of energy, Sensational: Body pains. Physiological: Headaches, Seizures, Heart beating fast & High blood pressure.

Themes under behavioural classification included observable behaviours like absentmindedness, isolation or social withdrawal, talking to oneself, crying, walking slowly, restlessness, and changes to sleeping patterns. These locally observed behaviours reflect emic understandings of depression, grounded in visible and socially interpreted signs. Through Kleinman’s lens, such expressions of distress are culturally meaningful and may differ from etic, clinical symptom checklists, highlighting the importance of context in diagnosis and care. A participant described a depressed young person saying:

*They are always absent-minded.* (Ghana FGD with high school boys’ participant)

A lived experience young person shared their experiences with absent-mindedness.

*I can be sitting here, but my mind is not here; I can be standing if someone doesn’t tap me, I can be standing in the sun.* (Young person with mental health lived experience, Ghana)

Crying was also frequently mentioned, with one young person stating:

*I normally find it difficult to deal with that* [stressful life events]*, so it leads to crying and other stuff.* (Young person with mental health lived experience, Ghana)

And another participant added that:

*My heart feels burdened, so that some other times I shed tears.* (Young person with mental health lived experience, Zimbabwe)

Social withdrawal was a common symptom, as highlighted by one participant:

*They* [depressed young people] *do not want to get closer to their friends, and when others are having fun, they sit alone.* (Ghana FGD with high school girls’ participant)

Another young person shared:

*I always go to a place where no one is there, and I will share my tears with the air and the trees. I don’t share anything with any human being, I keep things to myself.* (Young person with mental health lived experience, Ghana)

Two participants also explained that:

*You might find out that a child is always quiet at home, isolating themselves and not even engage with others.* (Young person with mental health lived experience, Zimbabwe)

*They are just people who usually escape into their own world.* (High school student, Zimbabwe)

Sleep disturbances were also reported, ranging from insomnia to hypersomnia. As one young person explained:

*Sometimes, when I am always in bed worried and thinking, I don’t always sleep.* (Young person with mental health lived experience, Ghana)

while another participant noted:

*Sleeping too much, that is the depression.* (Ghana FGD with high school girls’ participant)

Emotional symptoms of depression were thought to include pervasive worry and sadness. Participants stated:

*When someone is depressed, the person is always worried and does not know what to do.* (Ghana FGD with high school girls’ participant)

*Depression is the mood of a person or a disorder that causes sadness.* (Ghana FGD with high school girls’ participant)

*This is a medical situation when one feels lonely, sad, and unhappy about his or her life.* (Ghana FGD with high school boys’ participant)

Anger and mood swings were also reported, with participants mentioning that:

*The person is always angry and frustrated over a small issue.* (Ghana FGD with high school girls’ participant)

*You are in the mood and for no reason you just wake up angry for no reason again.* (Ghana FGD with high school girls’ participant)

Cognitive symptoms of depression were said to include hallucinations and suicidal thoughts, possibly pointing towards severe depression and/or psychotic depression, a subtype of major depressive disorder characterised by visual and auditory hallucinations as well as delusions. One young person described delusions that accompany depressive episodes, noting that:

Struggles with suicidal ideation were also brought up as another defining characteristic of depression. A focus group discussion participant commented that:

*When you have depression, everywhere the person goes, the person repeats, ‘I feel like dying.’ The person tries to commit suicide each and every time that something hurts him or her.* (Ghana FGD with high school boys’ participant)

Overthinking and an inability to concentrate were also common. One young person explained,

*You will think too much, so it will be like a burden on you*. (Young person with mental health lived experience, Ghana)

And another participant noted difficulty concentrating in class.

*Like difficult learning like this...sometimes, if I’m sitting in class, my mind is not always there.* (Young person with mental health lived experience, Ghana)

These accounts reflect emic interpretations of mental distress, where suicidal ideation are understood through personal and cultural lenses. Kleinman’s model helps frame these symptoms not only as clinical markers but also as expressions of deep psychological and social suffering, which may be interpreted differently in etic, biomedical contexts.

### Help-seeking behaviours in young people with depression

Help-seeking behaviours for depression among young people in Ghana and Zimbabwe include spiritual practices, family support, school mental health services, self-help strategies, and social networks (see Tables 4, 5). Notably, participants’ views on the causes and suitable treatment of depression often depended on their roles or professions. For example, community leaders and gatekeepers, such as faith and traditional healers, typically believed depression was caused by spiritual attacks. In contrast to traditional and faith healer beliefs about the explanatory models of depression and suitable treatments, health workers, including nurses and school counsellors, viewed depression as a medical issue linked to physical and psychological distress. Interestingly, young people’s help-seeking behaviours were closely tied to general beliefs about the causes and presentation of depression, which in turn were also influenced by a combination of cultural, traditional, and biomedical illness explanatory models of depression held by significant figures in the lives or environment. These contrasting perspectives reflect both emic and etic understandings of illness, where local cultural beliefs coexist with biomedical interpretations. Kleinman’s model helps illustrate how these diverse explanatory frameworks shape help-seeking behaviours, with young people often navigating between them based on the influence of significant figures in their lives and environments.

**Table 4:** Help-seeking amongst Ghanaian and Zimbabwean young people with depression.

Help seeking	
Category	Themes
**First point of call**	Public health services: (hospital), School health services: (Young person health clinic, School health master, school nurse), Spiritual care: (Church, Pastor/Spiritual leader, Pray to God, Traditional healer)
**Formal Care**	Nature of care: Health education (Medication – Pharmacy), Talk therapy, Negative Appraisal: Lack of patient monitoring, Negligence, Side effects Providers: School health master, Pastor, or Spiritual leader
**Informal Care**	Nature of Care: Economic Support, Self-help, Spiritual support, prayer Negative Appraisal: judgmental Providers: Friends and community members Spiritual healing: listening to religious sermons

**Table 5:** Characteristics of social support available to Ghanaian and Zimbabwean young people with depression.

Help seeking	
Category	Themes
**Family Support**	Nature of care: **Advice, Food, Personal care support,** accompany to seek care, Spiritual support, financial support Negative appraisal: inadequate care or concern from family Positive appraisal: conflict resolution Providers: Mother, Father, Brother
**Social Networks**	Nature of care: **Talk therapy, Singing,** Storytelling, Empathy, Financial or material support, Advice Forms of Social networks: **Peers/Friendship,** Family members, Nongovernmental Organizations, Teachers, **Community members**

### First point of call when seeking help with depression

Young people’s explanatory models of depression were closely linked to their caregivers’ or guardians’ beliefs and the environments they were in, such as schools with access to a chaplain for spiritual support. Spirituality was shown to play a significant role in understanding both the metaphysical nature of depression and its treatment. Reflecting an emic understanding shaped by cultural and spiritual worldviews, where depression is often interpreted through a spiritual lens, with young people sharing that:

*I am always saved by my God anytime I’m in trouble“* (Young person with mental health lived experience, Ghana)

*I normally pray to God.“* (Young person with mental health lived experience, Ghana)

A participant recounted how her aunt explained what was causing her depression, explanations that later influenced their help seeking behaviours.

*I called my mother’s sister, and she said she would roam* [seek spiritual guidance] *and see if there is something wrong someplace. so, I was told that my stepmother’s mother is the one who is doing all those things* [causing spiritual attacks]. (Young person, Ghana)

This reliance on faith is reinforced by family beliefs as a participant shared:

*In everything that happens, my mum always says that we should leave it to God.* (Young person with mental health lived experience, Ghana)

In contrast to young people’s beliefs about depression treatment, school health professionals like high school nurses and health tutors in Ghana incorporated both biomedical models, which focused on physical symptoms of depression and cultural beliefs, in their approach to address depression among young people attending senior high schools. For example, nurses noted that physical complaints like headaches and body pains often mask underlying emotional issues, such as relationship problems. This dual approach reflects an intersection of etic and emic perspectives, where clinical knowledge is blended with culturally informed understandings. Kleinman’s model helps illustrate how health professionals navigate these explanatory frameworks to provide care that resonates with both medical and local interpretations of distress.

*Sometimes we send them to the lab to do further investigation, at the end, you look at the whole thing, but the headache is there, then you will get to know that they are actually having issues and some too are having issues with their boyfriends, so they have headaches, sometimes general body pains, that is what I get.* (High School Nurse, Ghana)

High school health tutors in Ghana emphasised the challenge of addressing students’ beliefs in supernatural causes of their symptoms, such as witchcraft, and the need to demystify these beliefs. They also highlighted the role of spirituality in treatment, noting that praying with students who believe in spiritual solutions can provide comfort, recognising that depression could involve both medical and spiritual aspects. These insights reflect the coexistence of emic beliefs, rooted in cultural and spiritual understandings, and etic approaches grounded in biomedical models. Kleinman’s framework underscores the importance of acknowledging both perspectives, suggesting that concurrent spiritual and medical support may be more effective, especially as young people’s explanatory models are often shaped by the beliefs of influential figures in their environment.

*I remember there was a time we had to pray with them in the girls’ dormitory, we had to come with the Pastors to pray.* (High school Health tutor, Ghana)

*When they come, you try to talk to them to try to understand that it’s not true, and they try to say it’s spiritual or it’s witches and wizards that are disturbing them. So sometimes you have to demystify that witches and wizards are not in the dormitory, and sometimes, because they believe it’s spiritual, you have to pray with them. Especially those who believe prayers work when you pray with them, it’s fine.* (High school Health tutor, Ghana)

*Sometimes, they will sit down and be weeping, you will ask them, and they won’t talk. So, if you are experienced, you will know that they are depressed.* (School Housemaster, Ghana)

*So sometimes you have to talk to them or to advise them that it’s not true that there are witches or wizards, and sometimes when it is critical, you have to tell them to go to the hospital but at times, most of them want to go home.* (High school Health tutor, Ghana)

Additionally, health tutors mentioned the importance of advising students to seek medical help, when necessary, despite some students’ preference to return home. Showing the importance of culturally sensitive interventions that respect local beliefs while promoting effective mental health care practices.

Family support was also shown to be a crucial first point of call for young people who live with their caregivers, parents of guardians. The nature of support provided often comes in the form of advice. As one young person shared,

*My father always talks to me that I should avoid myself* [distance myself] *from people* [with bad influence] *so that it will not push me into trouble*. (Young person with mental health lived experience, Ghana)

While another mentioned:

*A sister or brother’s advice to stop worrying all the time and things will be better.* (Young person with mental health lived experience, Ghana)

School mental health services provided an essential first point of contact for boarding school students who spend the greater part of the year away from their family. One participant explained how the school health service facilitates her access to medications bought by her family.

*I first went to the clinic and the school health master. In school here the health master is the one mostly I say my drugs are finishing, so if you could call my mother and tell her to get drugs for me*. (Young person with mental health lived experience, Ghana)

Another noted how health talks provided at school are improving their mental health, stating that:

*They started it in Junior High but now it is called young person health, so we always go there and have our free chat with the nurses over there.* (Young person with mental health lived experience, Ghana)

#### Self-help strategies.

Self-help strategies for depression were varied and include activities such as singing, exercising, and writing. young people with lived experience described their experiences mentioning that:

*If something happens to me and I am annoyed, I always sit down while singing gospel or songs that will encourage myself.* (Young person with mental health lived experience, Ghana)

*On Saturdays we go to the chapel for choir practice, and I am one of the choir masters. So when we are there, I don’t really get distracted.* (Young person with mental health lived experience, Ghana)

*Going to church will make you happy, in the church, your friends and family might be there, and you feel connected to them.* (Young person with mental health lived experience, Ghana)

While others shared that:

*Sometimes when you are singing, you forget some things and it will change your mood and makes you happy.* (Young person with mental health lived experience, Ghana)

Physical activities and journaling also served as relaxation technics, as participants noted:

*I go to a free space and cry them out, or I will write it in a book, or I will sing. These are things, I use to deal with my mental issues but even at times when I’m in the house. I will go out and jog out like that.* (Young person with mental health lived experience, Ghana)

*Sometimes I feel like doing some exercise or I will just go and have fun with my friends and chat before I fetch my water and go and bath.* (Young person with mental health lived experience, Ghana)

One young person mentioned how spending time alone helped with their depression:

*I only help myself by avoiding people. I will just go somewhere far away where no one is closer to me, that way I can have peace of mind.* (Young person with mental health lived experience, Ghana)

These practices reflect emic approaches to managing distress, rooted in personal and culturally meaningful coping mechanisms. Through Kleinman’s lens, such strategies highlight how individuals draw on familiar, accessible resources to restore emotional balance, often outside formal medical systems. While these may not align with etic clinical interventions, they offer valuable insights into how young people navigate their mental health within their own cultural and social contexts.

#### Support networks.

Participants shared various forms of social support available to young people with depression in Ghana and Zimbabwe, emphasizing both family and social networks (see Table 5). Family support encompasses practical and emotional care, including advice, food, personal care, spiritual support, and financial assistance, primarily provided by close relatives such as parents and siblings. However, there are instances of inadequate care or concern that negatively impact young people’s mental health. Social networks extended beyond the family, offering informal talk therapy, singing, storytelling, empathy, and material support through peers, extended family members, school staff, and the general community. These varied sources of support reflect emic understandings of healing and care, grounded in relational and communal values. Kleinman’s model highlights how these culturally embedded practices can complement etic, clinical approaches, underscoring the importance of a multifaceted mental health care strategy that integrates both familial and community resources to effectively address the needs of young people with depression.

Financial support in the form of money, food and school supplies, spiritual support like prayers as well as support with medical appointments like being accompanied by a caregiver or friends to seek help topped the list. However, some young people felt neglected or having to rely on well-wishers to provide for their needs which became a source of worry and embarrassment especially were peers in school come from and economically health backgrounds. As one participant noted:

*There is this friend I have in the dormitory he is actually my junior and he is helping me with this condition. He says he himself faces it, and he tell himself when to do this and when not to do that.* (Young person with mental health lived experience, Ghana)

while others emphasized the importance of peer advice as an important part of depression help seeking.

*I just said I will stop schooling and just be free and she* [friend] *just advised me that I should continue.* (Young person with mental health lived experience, Ghana)

*I think we should talk to them and try and help as peers before we tell our elders.* (High school student, Zimbabwe)

These experiences reflect emic perspectives on care and suffering, where material and spiritual support are deeply intertwined with emotional well-being. Kleinman’s model helps frame these accounts as culturally meaningful expressions of distress and healing, which may not be fully captured by etic, biomedical frameworks that overlook the social and economic dimensions of mental health.

While many explanatory models and help-seeking behaviours were shared across Ghana and Zimbabwe, some country-specific differences emerged. In Zimbabwe, emic perspectives were more strongly shaped by idioms like ‘kufungisisa’ and spiritual explanations involving ancestral spirits and witchcraft. Young people often described depression as “thinking too much” and sought help from spiritual leaders or the Friendship Bench programme. In Ghana, emic expressions such as ‘taamebubasugbor’ were less frequently used, and spiritual beliefs were more often linked to religious practices and moral transgressions. Etic perspectives in both countries included references to sadness, suicidal thoughts, and academic stress, but Ghanaian participants more frequently discussed school-related pressures and economic hardship, while Zimbabwean participants emphasised interpersonal rejection and stigma related to HIV. These differences highlight the importance of tailoring interventions to local cultural and social contexts.

## Discussion

This study explored the explanatory models of depression among young people in Ghana and Zimbabwe, incorporating perspectives from young persons, caregivers, teachers, healthcare workers, community leaders, and policymakers. The findings highlight significant cultural, socio-economic, and individual factors influencing the understanding, manifestation, and help-seeking behaviours for depression in these contexts. Highlighting the importance of understanding the socio-economic, cultural, and individual factors shaping young people’s mental health experiences to address the depression treatment gap effectively. Reinforcing conclusions made by other researchers that depression remains largely untreated in low-resource settings due to stigma, limited resources, and cultural beliefs [6,25] especially among young people.

Using the Emic and Etic framework, this study reveals both insider (emic) and outsider (etic) perspectives on depression. Emically, participants described depression in culturally resonant terms such as “thinking too much” or having a “heavy heart,” reflecting traditional understandings of emotional distress in Zimbabwe, Ghana and similar Sub Saharan African contexts, resonating with observations made in both recent systematic reviews on young people as well as evidence from clinical trials with adults with depression [22,25,26]. Supernatural explanations, such as witchcraft or curses, were also brought out, emphasizing the role of spiritual frameworks in shaping perceptions of illness. These beliefs often led to spiritual interventions as the first point of call, such as prayer and seeking help from spiritual leaders. Etically, the study identified socio-economic stressors, interpersonal challenges, and biological vulnerabilities as key contributors to depression, aligning with global understandings of the condition [1].

Kleinman’s Explanatory Models of Illness framework [14] helps to contextualise the diverse explanations for depression found in this study. Participants’ perceptions revealed a multilayered understanding of depression, integrating socio-economic factors like poverty, unemployment, and academic pressure. Which are reflective of the precarious socio-economic conditions prevalent in African countries, further cementing Olisaeloka et al [2024]’s point that treatments developed in high-income countries are not easily transferable for use in LIMCs due to the differences in socioeconomic contexts [25]. Interpersonal stressors, including familial conflicts, peer rejection, and bereavement, were significant contributors to depression, consistent with evidence that relationship crises or loss can precipitate depression in vulnerable young people [11,27]. These explanatory models highlight the need for interventions that bridge traditional and biomedical approaches. The initial conceptualization of depression often involves supernatural perspectives and the influence of peers, caregivers, and significant people in young people’s lives, even when a socioeconomic cause has been identified. This is compounded by young people’s personal struggles with accepting the reality of mental health disorders and the societal stigma attached to mental illness, aligning with the observations reported in other studies [1]. These factors have been shown to delay formal help-seeking from biomedical services.

In terms of individual manifestations of distress, participants described symptoms of depression across behavioural, emotional, cognitive, and physical domains, reflecting culturally embedded understandings of distress consistent with Kleinman’s Explanatory Model of Illness. Behavioural symptoms, such as social withdrawal and frequent crying, were especially visible in school settings, where they often coincided with declining academic performance. Emotional symptoms, including persistent sadness and worry, were frequently linked to social stressors like bullying and body shaming, highlighting perceived causes rooted in interpersonal experiences. Physical complaints, such as headaches and fatigue, illustrated the somatic expression of distress commonly observed in African contexts, where emotional suffering is often communicated through bodily symptoms [1,14]. Underscoring how young people’s explanatory models integrate both psychological and somatic dimensions, shaped by their social environments and cultural norms.

Within interpersonal contexts, help-seeking behaviours demonstrated a strong reliance on informal and communal support systems. Spiritual leaders, family members, and peers were frequently the first points of contact, illustrating how social networks shape understandings of illness and influence treatment pathways. This pattern aligns with Kleinman’s Explanatory Model of Illness, which emphasises the role of culturally embedded beliefs in shaping perceptions of causation and appropriate responses to distress. In many cases, depression was interpreted through spiritual frameworks, such as curses, spiritual attacks, or moral transgressions, which guided individuals toward spiritual healing practices. While these approaches were often perceived as effective or satisfactory within the community, they could also delay engagement with biomedical services.

The Emic/Etic framework further illuminates these dynamics. Emic perspectives, grounded in local cultural understandings, framed depression in terms of spiritual imbalance or social disharmony, reinforcing the legitimacy of traditional and spiritual support systems. In contrast, etic perspectives, rooted in biomedical models, conceptualised depression as a clinical condition requiring formal mental health care. The coexistence of these explanatory models reflects the pluralistic nature of health systems in many African settings. However, the lack of consensus in the literature regarding whether biomedical or spiritual models should be prioritised highlights the complexity of navigating these pluralistic systems. Kleinman’s framework underscores the importance of recognising and respecting these coexisting models, as they reflect deeply held cultural paradigms and significantly influence both the trajectory of illness and the acceptability of interventions.

Findings from the present study showed that schools played a vital role in identifying, demystifying and addressing depression but limited mental health literacy among educators and caregivers created gaps in support. Evidenced by depression being often referred to more broadly as mental illness, accompanied by the general stigma associated with conditions such as schizophrenia, thereby discouraging young people from seeking help due to fear of being labelled or stigmatized. This finding is in line with previous studies in Zimbabwe and other African countries [16,26,28] observing that depression, in its current Eurocentric definition does not quite translate well in Africa communities where notions of ‘a heavy heart’ or ‘thinking too much’ encapsulate what depression is.

A major departure of this study’s findings from existing literature is that young people’s explanatory models of depression appeared to be two-pronged. On one hand, these models were heavily influenced by the beliefs about depression held by the people around them or the environment they were in. For example, young people in missionary-Christian schools believed in spiritual explanations and the power of prayer, while those from other religious backgrounds believed in alternative forms of spiritual support. Others believed in the efficacy of biomedical treatments, such as taking mental health medication. On the other hand, young people’s explanatory models were shaped by personal lived experiences of hardship, perceived betrayal, financial neglect, and adversity, which they saw as causes of depression beyond their control. These experiences included forced marriage, coming from a poor family, peer pressure, bullying, and discrimination. The subjective nature of these experiences and the tendency of adults to overlook young people as experts by experience often result in sidelining young people as active participants in finding solutions or treatment for their own depression, favouring socially prescribed understandings of depression and socially desirable help-seeking behaviours.

To address the identified challenges, culturally sensitive interventions are essential. The Friendship Bench in Zimbabwe, which adapts evidence-based therapies to local contexts, demonstrates the feasibility of scalable mental health solutions [27]. Expanding such models to address youth-specific challenges, such as academic stress and early marriages, could enhance their impact. Leveraging community structures, such as schools and faith-based organisations, offers practical pathways for intervention delivery. Training educators, caregivers, and lay health workers to identify and respond to depression can bridge gaps between traditional and biomedical systems, reducing stigma and improving treatment adherence.

Policy-level interventions are crucial to support the integration of mental health services within existing healthcare and educational systems. Governments and stakeholders should prioritise funding for mental health resources and training programs that equip frontline workers with the skills to recognize and manage depression among young people. Collaborative efforts between governmental bodies, non-governmental organisations, and community leaders can foster an environment conducive to depression awareness and support, thereby enhancing the reach and effectiveness of interventions.

One of the strengths of this study is its comprehensive approach, incorporating multiple perspectives from various stakeholders, which provides a holistic understanding of depression among young persons in Ghana and Zimbabwe. The use of qualitative methods allowed for in-depth exploration of participants’ experiences and beliefs, offering rich, contextual insights. However, One limitation of this study is its reliance on self-reported data, which may be influenced by social desirability or stigma, especially in discussions of sensitive topics like depression. To mitigate this, interviews were conducted in private settings by trained research assistants using trauma-informed approaches, and participants were assured of confidentiality and their right to withdraw at any time. Another limitation is the geographic scope, which focused on specific regions in Ghana and Zimbabwe. While this limits generalisability, the purposive sampling strategy and inclusion of diverse stakeholder groups helped ensure contextual richness and relevance. Additionally, the use of bilingual research assistants and culturally adapted topic guides helped reduce misinterpretation and enhance the validity of responses.

### Implications for future research

This study highlights several context-specific gaps that future research should address. First, interventions should be designed to reflect the hybrid explanatory models held by young people—combining biomedical language with spiritual and cultural idioms such as kufungisisa and taamebubasugbor. Research should explore how these hybrid models influence treatment engagement and outcomes, particularly in youth populations.

Second, given the prominence of school-related stress and the role of educators in identifying distress, future studies should evaluate the effectiveness of school-based mental health literacy programmes tailored to local idioms and stigma patterns. This includes assessing how teachers and school nurses can be trained to recognise culturally framed symptoms and refer students appropriately.

Third, the reliance on peers and informal networks suggests a need to investigate peer-led interventions. Research should examine how peer support groups or youth ambassadors can be integrated into community-based mental health strategies, especially in settings where formal services are limited.

Finally, future studies should explore how to engage traditional and faith-based healers in collaborative care models. Given their influence on help-seeking behaviours, understanding how these actors can support or hinder formal mental health integration is critical. Longitudinal research could also examine how explanatory models evolve over time and across different youth subgroups, informing scalable and sustainable intervention designs.

## Conclusions

This study contributes to the growing literature on youth mental health in low-resource settings by offering a culturally grounded analysis of how young people in Ghana and Zimbabwe conceptualise depression. Using Kleinman’s Explanatory Model and the emic/etic framework, the study reveals that young people often hold hybrid explanatory models, combining biomedical symptom descriptions with spiritual and culturally embedded beliefs. These models shape help-seeking behaviours and influence the acceptability of mental health interventions. A key contribution of this study is its multi-stakeholder approach, incorporating perspectives from young people, educators, caregivers, and health workers to inform contextually relevant intervention design. The findings underscore the importance of integrating local idioms of distress, spiritual beliefs, and informal support networks into youth mental health strategies.

However, the study’s scope was limited to two regions, Harare in Zimbabwe and Navrongo in Ghana, which may affect the generalisability of findings. While the explanatory models identified may resonate with other low-resource settings, caution is needed in applying these insights beyond the study contexts. Future research should explore how explanatory models evolve across different youth subgroups and settings, and how culturally adapted interventions can be co-designed with communities. Policymakers and practitioners should consider hybrid care models that bridge traditional and biomedical systems, and invest in school-based and peer-led mental health support tailored to local realities.

## Supporting information

S1 File Inclusivity in global research. (DOCX)
